# Atypical Adult-Onset Still’s Disease Presenting With Seizures and Myocarditis Complicated by Macrophage Activation Syndrome

**DOI:** 10.7759/cureus.52635

**Published:** 2024-01-20

**Authors:** Anh N Le, Nino Gudushauri, Irene J Tan, Rekha Bhat, Rasha Khan

**Affiliations:** 1 Internal Medicine, Philadelphia College of Osteopathic Medicine, Philadelphia, USA; 2 Internal Medicine, Einstein Medical Center Philadelphia, Philadelphia, USA; 3 Rheumatology, Einstein Medical Center Philadelphia, Philadelphia, USA; 4 Pathology and Laboratory Medicine, Einstein Medical Center Philadelphia, Philadelphia, USA

**Keywords:** adult-onset still’s disease (aosd), recurrent seizure, seizure, right ventricle failure, myocarditis, hyperferritinemia, organomegaly, macrophage activation syndrome (mas), hemophagocytic lymphohistiocytosis (hlh)

## Abstract

Adult-onset Still’s disease (AOSD) is a rare multi-systemic inflammatory disorder characterized by high spiking fevers, nonpruritic, salmon-colored rash, and severe polyarthralgia. Laboratory features typically include elevation in white blood cells, liver enzymes, and ferritin. Central nervous system and cardiac involvements, particularly myocarditis, are rare. Macrophage activation syndrome (MAS) is a well-described complication of AOSD, leading to a high mortality rate. Herein, we describe a case of AOSD complicated by MAS in a 32-year-old male presenting with atypical clinical manifestations, including recurrent seizures, scaly, pruritic, and hyperpigmented rash, and right heart failure due to lymphocytic myocarditis. The patient exhibited a delayed onset of fever, leukocytosis, and transaminitis that initially deterred eligibility for Yamaguchi criteria for AOSD. Bone marrow and lymph node biopsies did not show malignancy, infection, or hemophagocytosis. However, soluble interleukin-2 receptor alpha or soluble CD-25 was elevated. The patient experienced significant improvement on combination therapy of anakinra, methotrexate, and stress-dose steroids. HScore was later indicative of a high probability for MAS. Outpatient management involved prednisone, cyclosporine, and canakinumab for MAS. Seizure and myocarditis are possible presenting features of atypical AOSD. Early recognition of non-criteria AOSD and MAS and prompt initiation of therapy may prevent mortality.

## Introduction

Adult-onset Still’s disease (AOSD) is a rare inflammatory disorder with multiorgan involvement. The etiology of AOSD remains unknown since it was first classified as a distinct clinical entity in 1971. The hallmark clinical features of AOSD include high spiking fevers, evanescent, salmon-colored rash, and severe polyarthralgia [1]. Laboratory investigation typically features leukocytosis, elevated liver enzymes, and elevated ferritin. Reports are scarce in AOSD patients presenting beyond these organ systems. Cardiac, particularly myocarditis, and central nervous system manifestations in AOSD are exceedingly rare, accounting for 5-7% [2,3] and 7-12% [4] of all AOSD cases, respectively. Hemophagocytic lymphohistiocytosis (HLH) occurs when the immune system goes awry causing both macrophages and T-lymphocytes to activate continuously, resulting in an exuberant inflammatory cytokine storm [5,6]. When HLH is triggered by an underlying autoimmune disorder, such as AOSD or systemic lupus, it is termed macrophage activation syndrome (MAS) [5]. The mortality rate of patients with AOSD is 9.5% [7]. However, mortality can be as high as 52.9% if AOSD is complicated by MAS [8]. In this report, we describe a complex case of AOSD complicated by MAS who presented with recurrent seizures, an atypical rash, initial laboratory aberrancy, and severe right heart failure secondary to lymphocytic myocarditis.

## Case presentation

**Figure 1:**
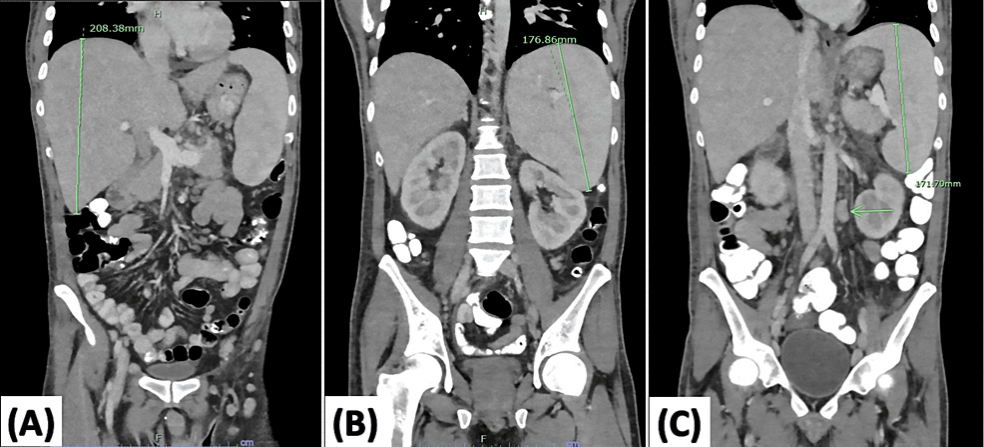
Coronal views of computed tomography (CT) of the chest, abdomen, and pelvis with contrast were completed during the first hospital encounter. (A) New hepatomegaly in the right lobe measuring 21 cm (previously 16 cm). (B) New splenomegaly measuring 17.7 cm (previously 8.9 cm) and bilateral nephromegaly (measurements not shown; left kidney 12.9 cm and right kidney 12.6 cm, both previously 11.3 cm). (C) Para-aortic lymphadenopathy (indicated by the green arrow, measuring >2 cm).

**Figure 2:**
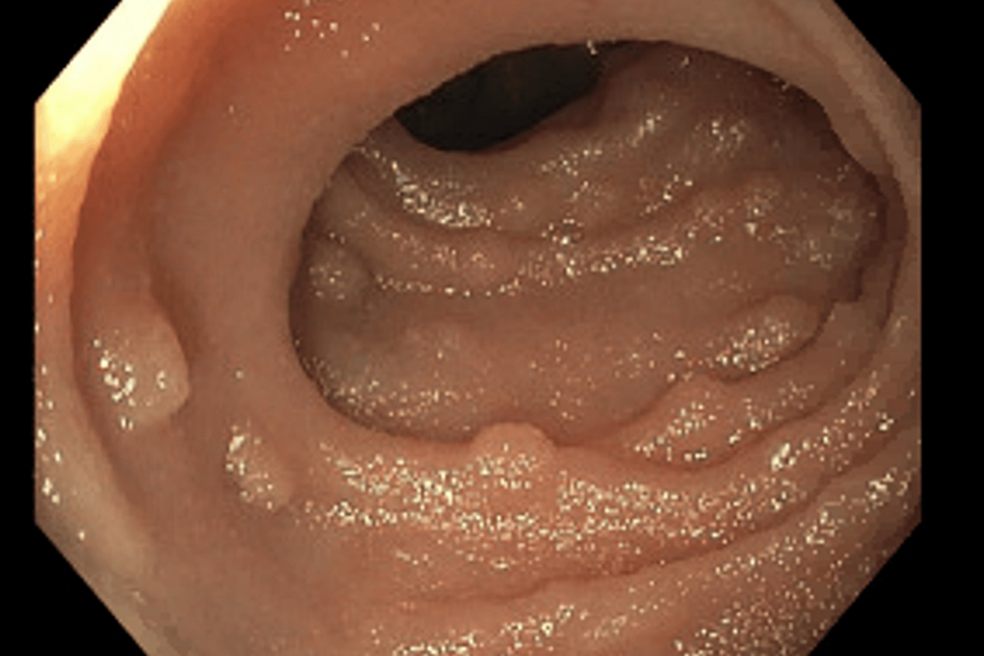
A colonoscopy performed due to bright red blood per rectum during the first hospital admission revealed nodular ileal hyperplasia.

A 32-year-old man with no prior medical history had two consecutive hospital admissions six weeks apart. He took no prescription or over-the-counter treatments. He denied past or current use of cigarettes, alcohol, or illicit drugs. He is of East Asian descent but was born and raised in the United States. He denied sick contacts or a history of travel for most of his adult life. At the initial admission, he presented with seizure-like activity with subsequent loss of consciousness and bright red blood per rectum. He was noted to have extremely elevated ferritin, leukocytosis, eosinophilia, severe microcytic iron and folate deficiency anemia, and normal platelet counts (Table 1). Computed tomography (CT) of the head without contrast did not show any acute intracranial hemorrhage, midline shift, or mass effect. When compared to a scan from three years prior, contrast-enhanced CT of the chest, abdomen, and pelvis revealed new organomegaly with hepatomegaly (right lobe 21 cm, previously 16 cm), splenomegaly (17.7 cm, previously 8.9 cm), and bilateral nephromegaly (left kidney 12.9 cm and right kidney 12.6 cm, both previously 11.3 cm), as well as lymphadenopathy in the hepatic portal region, mesentery, retroperitoneum, pelvis, and inguinal areas (Figure 1). Colonoscopy revealed a nodular patchy area of the terminal ileum; biopsy of which showed small intestinal mucosa with prominent lymphoid aggregates (Figure 2). Routine electroencephalogram (EEG) when awake and asleep completed four days after the presentation was normal. The neurology consultant determined that his presenting seizure-like activity was likely convulsive syncope secondary to anemia. He received a transfusion with one unit of packed red blood cells, and hemoglobin (Hgb) was 7.5 gm/dL on discharge (Table 1). He was discharged with a hematology-oncology outpatient follow-up to address anemia with organomegaly and hyperferritinemia.

**Table 1:** Selected laboratory results and interventions over two hospital admissions and ambulatory clinic follow-up. HD = hospital day; DC = hospital discharge; s/p = status post; WBC = white blood cell count; Hgb = hemoglobin; PLT = platelets; ESR = erythrocyte sedimentation rate; CRP = C-reactive protein; ALT = alanine transaminase; AST = aspartate aminotransferase; LDH = lactate dehydrogenase; IL-2R⍺/sCD25 = soluble interleukin-2 receptor alpha or soluble CD25; RR = reference range; MCV = mean corpuscular volume; CMV = cytomegalovirus; EBV = Epstein-Barr virus; anti-VCA = viral capsid antigen antibody; anti-EA = early antigen antibody; anti-EBNA = Epstein-Barr nuclear antigen antibody; ACE = angiotensin-converting enzyme; CCU = cardiac care unit; SDU = step down unit; MICU = medical intensive care unit; SC = subcutaneous.

	WBC (4-11 x 10^3^/uL)	Hgb (14-18 g/dL)	PLT (112-361 × 10^3^/uL	ESR (0-15 mm/hr)	CRP (0-5 mg/L)	Ferritin (22-275 ng/mL)	Creatinine (0.7-1.2 mg/dL)	ALT (0-55 IU/L)	AST (5-34 IU/L)	LDH (125-220 IU/L)	IL-2R⍺/sCD-25 (532-1891 pg/mL)	Other laboratory values	Transfers & treatments
Initial admission													
HD #1	14.3	6.8	319	81	73	34,120	0.4	<5	24			MCV: 74.8 fL (RR: 81-96); iron: 22 mcg/dL (RR: 65 -175); folate: 3.1 ng/mL (RR: 5.4-20); absolute eosinophil count: 1.1 x 10^3^/mcL (RR < 0.6)	General medicine unit – transfusion, intravenous fluid
HD #4	9.6	8	364			31,492	0.45	<5	18	617			Iron, folate
HD #5	10.3	7.5	504				0.4	<5	22				DC to home with hematology/oncology outpatient follow-up
Second admission													
HD #1	7.6	6.6	316	90	98	35.380	0.56	<5	26	936		MCV: 78.8 fL (RR: 81-96); reticulocyte count: 3.86% (RR: 0.6-2.2); albumin: 1.5 g/dL (RR: 3.5-5.0); globulin: 6.1 gm/dL (RR: 2.1-3.7); alkaline phosphatase: 235 U/L (RR: 40-150); fibrinogen: 422 mg/dL (RR: 191-491); triglyceride: 256 mg/dL (RR: 0-150); CMV IgG >10 AU/mL (RR < 0.6); CMV IgM 44.2 AU/mL (RR < 30); EBV anti-VCA IgG 144 U/mL (RR < 18); EBV anti-EA 12.9 U/mL (RR < 9); anti-EBNA: 24.3 U/mL (RR < 18); ACE: 80 U/L (RR < 67)	General medicine unit – transfusion, iron, folate, levetiracetam
HD #4	5.7	6.8	373	61	110	>40,000	0.53	<5	23	938			Transfusion
HD #5	10.2	7.8	402	70	131	39,111	0.62				7,401		CCU – pressors, anakinra 100 mg SC every 8 hours
HD #7													SDU – tapered pressors. Missed 2 doses of anakinra following transfer
HD #9	4.2	6.5	449	62	23	>40,000	0.5						Transfusion
HD #10	5.9	8.1	445	51	20	31,488	0.6						Missed 2 doses of anakinra
HD #11	6.7	7.7	450	84	24	35,027	0.53	6	34				Missed 1 dose of anakinra
HD #12	11.3	7.7	427					6	31				MICU – restarted pressors. Initiated stress-dose hydrocortisone 50 mg every six hours. Restarted anakinra 100 mg SC every 8 hours
HD #13	18	8.4	446	85		>40,000	0.4	10	62				Methotrexate 25 mg SC once, folic acid 1 mg daily
HD #15	2.9	7.3	336	90	33	33,511	0.45	10	55				Completed 3 days of stress dose hydrocortisone. General medicine unit
HD #16	1.7	7.6	339	73	17.3	39,589	0.5	15	48		13,209		Increased folic acid to 3 mg daily. Tapered anakinra to 100 mg SC daily
HD #17	2.3	7.5	369			17,211	0.44	15	39		6,069		
HD #19	4	8.2	417			7,750	0.49	16	28				DC to home with prednisone 25 mg daily taper, folic acid 3 mg daily
Ambulatory clinic													
5 days s/p DC	15	10.7	274	106	35	2,770	0.56	17	34				Prednisone 40 mg taper. Initiated cyclosporine 100 mg daily and canakinumab 300 mg SC every 4 weeks. Discontinued anakinra and folic acid
6 weeks s/p DC	9	12.5	251	53	6	576	0.82	17	24				Prednisone 15 mg taper. Continued canakinumab 300 mg SC every 4 weeks and cyclosporine 100 mg daily

**Figure 3:**
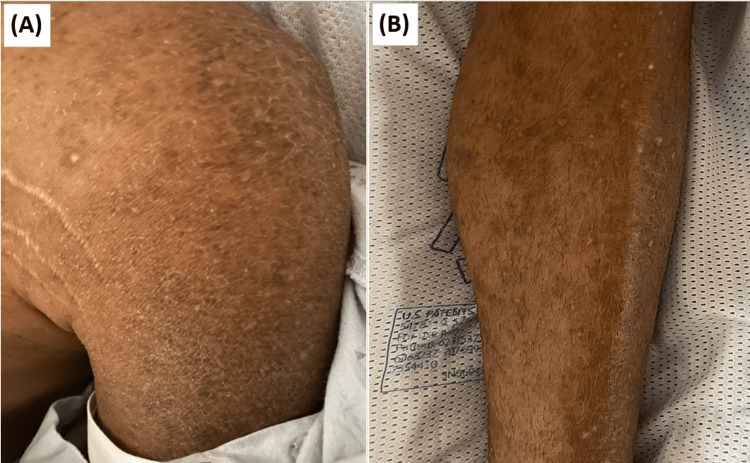
Photographs of the scaly, hyperpigmented, maculopapular rash over the (A) left shoulder and (B) right lower leg.

The subsequent hospital admission six weeks later was prompted by a witnessed tonic-clonic seizure while resting followed by loss of consciousness. He endorsed a 4 lb (1.8 kg) weight loss in one month, and the onset of diffuse and severe joint pain affecting the wrists, hips, knees, ankles, and toes with painful swelling in the knees and soles of the feet. This was followed by the eruption of a pruritic rash over all extremities and torso (Figure 3). He described a preceding upper respiratory tract infection with sore throat, chills, and night sweats without fever that had spontaneously resolved.

On arrival, he was afebrile with a temperature of 37°C, tachycardic with a heart rate of 106 beats per minute (bpm), hypotensive with a blood pressure of 88/47 mmHg, and pulse oxygen saturation (SpO2) of 98% on room air. Physical examination was remarkable for a diffuse, scaly, hyperpigmented rash on his trunk and all limbs, hepatosplenomegaly, diffuse lymphadenopathy, markedly tender, boggy synovitis in the wrists and knees, as well as bilateral tenderness on palpation of the metacarpophalangeal joints, proximal interphalangeal joints of hands, ankles, and second through fifth metatarsophalangeal joints of both feet. Initial investigation revealed hypoalbuminemia, microcytic anemia with elevated reticulocyte count, and significantly elevated inflammatory markers, namely, ferritin, erythrocyte sedimentation rate (ESR), and C-reactive protein (CRP). Additionally, he was found to have elevated alkaline phosphatase, elevated lactate dehydrogenase, and elevated fibrinogen with hypertriglyceridemia (Table 1). Head CT without contrast was unremarkable. EEG was normal on hospital day three while on levetiracetam.

Four broad categories of disorders were considered for this patient, namely, malignancy, infection, infiltrative diseases, and rheumatic diseases. Malignancy, lymphoproliferative diseases in particular, was on the top of the differential given the presentation of a young male with weight loss and extensive lymphadenopathy. He underwent a bone marrow (BM) biopsy and a right inguinal excisional lymph node (LN) biopsy to rule out malignancy and hemophagocytosis. BM biopsy and flow cytometry revealed normal cellularity with trilineage hematopoiesis, polyclonal plasmacytosis, and no evidence of leukemia, lymphoma, or plasma cell dyscrasia. BM and LN biopsies, as well as CT scans of the head and chest, abdomen, and pelvis, and colonoscopy, were negative for malignancy.

Secondly, extensive laboratory tests were performed with infectious disease consultation, which eventually ruled out active bacterial and viral infections (i.e., syphilis, infectious mononucleosis, human immunodeficiency virus (HIV), herpes simplex virus 1 and 2 (HSV1 and HSV2) infections, and mycobacterial infections), indolent fungal infections, and parasitic infections. Cytomegalovirus (CMV) IgG and IgM antibodies and Epstein-Barr virus (EBV) IgG antibodies were increased (Table 1). However, DNA polymerase chain reaction (PCR) tests for CMV and EBV were not detected, excluding active viremia. Screening was also negative for HIV, HSV1, HSV2, syphilis, and β-d-glucan (BDG) test. QuantiFERON Tuberculosis Gold test was indeterminate and deemed not worthwhile to repeat since chest CT did not show hilar adenopathy or parenchymal disease. The fungal stain on LN was negative for granulomas. The stool was negative for ova and parasites.

Thirdly, infiltrative diseases, such as amyloidosis, sarcoidosis, Rosai-Dorfman disease, Kikuchi-Fujimoto disease, IgG4-related diseases, and Castleman’s disease were also considered. Angiotensin-converting enzyme (ACE) level was slightly increased (Table 1). Serum IgG (3,259 mg/dL; reference range (RR) 600-1,640), IgG subclass 1 (2,177 mg/dL; RR 382-929), IgG subclass 3 (>220 mg/dL; RR 22-178), and IgG subclass 4 (102.4 mg/dL; RR 4-86) were elevated. However, LN biopsy did not support any of these diagnoses. Although LN biopsy did show interfollicular polyclonal plasmacytosis within IgG4 cells with IgG/IgG4-focal increase in IgG4 positive cells, the absence of the characteristic storiform patterns and IgG/IgG4 ratio of 35% did not meet criteria for IgG4-related diseases.

Lastly, rheumatologic disorders and immunologic disorders, such as systemic lupus erythematosus (SLE), AOSD, and MAS/HLH were entertained early on. Rheumatoid factor (RF), anti-nuclear antibody (ANA), anti-Smith, anti-ribonucleoprotein (RNP), anti-Ro, anti-La, anti-centromere, anti-Jo-1, anti-ribosomal RNP, and anti-neutrophil cytoplasmic (ANCA) antibodies were all negative per rheumatology consultation. Complement C3 and C4 were within normal limits. Anti-β2 microglobulin was mildly elevated (5.61 mg/L; RR ≤ 2.51). SLE was ruled out with negative ANA and normal C3 and C4 complements. AOSD and MAS were high on our differential diagnoses. Despite the presence of hepatosplenomegaly, lymphadenopathy, and negative tests for ANA and RF, his initial clinical constellation did not satisfy Yamaguchi’s classification criteria for diagnosis of AOSD due to the absence of high-grade fever, typical nonpruritic, evanescent rash, leukocytosis, or abnormal liver enzymes (Table 2) [7]. Concomitantly, the calculated HScore for reactive hemophagocytic syndrome estimated the probability of HLH at <18% given absences of known immunosuppression, fever, cytopenia in two or more lineages, transaminitis, and fibrinogen <250 mg/dL (Tables 1, 2) [6]. Atypical presentations of AOSD, including the late onset of several key features, delayed the diagnoses of these interrelated disorders.

**Table 2:** Yamaguchi classification criteria for adult-onset Still's disease, HScore diagnostic score for reactive hemophagocytic syndrome, and 2016 classification criteria for macrophage activation syndrome. AOSD = adult-onset Still's disease; MAS = macrophage activation syndrome; WBC = white blood cell count; HIV = human immunodeficiency virus; Hgb = hemoglobin; PLT = platelet count.

Yamaguchi’s criteria for AOSD - ≥5 criteria must be met, including ≥2 major criteria		HScore for reactive phagocytosis	2016 classification of MAS
Major criteria	Fever ≥ 39°C persisting for ≥1 week	Known underlying immunosuppression (HIV positive or receiving long-term immunosuppressive therapy)	
	Arthralgia/arthritis persisting for ≥2 weeks	Temperature ≥ 38.4°C	
	Typical rash	Hepatomegaly and/or splenomegaly	
	WBC ≥10×10^9^/L (>80% neutrophils)	Ferritin ≥ 2000 ng/mL	Ferritin ≥ 7000 ng/mL and any 2 of the below
Minor criteria	Sore throat	Cytopenia in ≥ 2 lineages (i.e., Hgb ≤ 9.2 g/dL, WBC ≤ 5,000/mm³, PLT ≤ 110,000/mm³)	PLT < 180,000/mm³
	Lymphadenopathy	Triglyceride ≥ 132.7 mg/dL	Triglyceride > 156 mg/dL
	Hepatomegaly or splenomegaly	Fibrinogen < 250 mg/dL	Fibrinogen < 360 mg/dL
	Abnormal liver function test	AST ≥ 30 U/L	AST > 50 U/L
	Negative IgM rheumatoid factor and antinuclear antibodies	Hemophagocytosis features on bone marrow aspirate	
Exclusion criteria	Infection (e.g., sepsis and infectious mononucleosis)		
	Malignancy (e.g., lymphoma)		
	Other rheumatic diseases (e.g., polyarteritis nodosa and vasculitis secondary to rheumatoid arthritis)		

**Figure 4:**
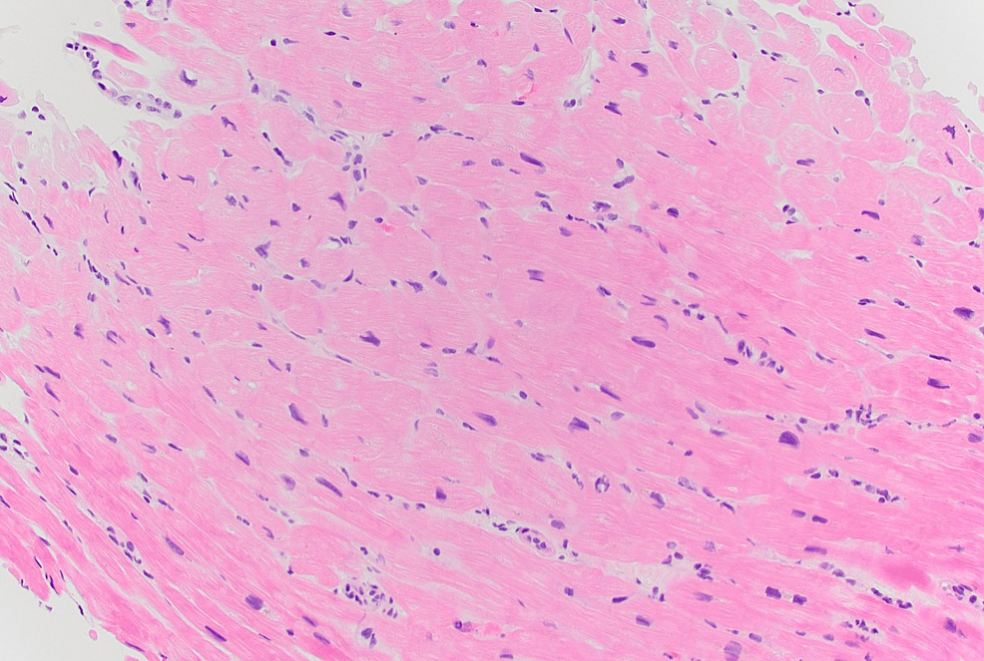
Hematoxylin and eosin-stained section (200x​) of right ventricle biopsy showing mild lymphocytic infiltrate.

During the second hospitalization, the patient was given fluid resuscitation and required a transfusion for symptomatic anemia. He was maintained on levetiracetam 500 mg every 12 hours for seizure prophylaxis, folic acid, and iron supplementation for anemia. On days four to five of admission, he developed new onset chest pain, cough, and shortness of breath with worsening sinus tachycardia (123 bpm), hypotension (72/37 mmHg), hypoxia requiring oxygen (SpO2 90% on 2L nasal cannula), and a spike in temperature to >38.4°C. Pulmonary embolus was ruled out via CT angiography of the chest, which demonstrated interval worsening of pulmonary edema. Transthoracic echocardiogram (TTE) showed a preserved ejection fraction of 60% and was severely dilated with moderate to severely reduced systolic function of the right ventricle (RV). The clinical course was complicated by acute-onset severe right heart failure (RHF) requiring transfer to the cardiac care unit (CCU) for pressor support. This was deemed out of proportion to the mild pulmonary arterial hypertension (PAH) seen via right heart catheterization (RHC). RHC findings yielded normal coronaries, right atrial pressure of 2 mmHg, right ventricle pressure of 35/3 mmHg, pulmonary arterial pressure (PAP) of 38/18 mmHg with mean PAP of 25 mmHg, mean pulmonary capillary wedge pressure of 6 mmHg. Brain natriuretic peptide (BNP) was elevated (490.6 pg/mL; RR: 0-100), and troponin-I was negative. The combination of fever, acute chest pain, elevated BNP, and focal RV wall abnormalities on TTE with signs of RHF raised clinical suspicion for myocarditis as a complication of AOSD. Endomyocardial biopsy of the RV septum revealed prominently vacuolated cardiomyocytes with scattered interstitial CD3 positive T-cell infiltrate (Figure 4). Empiric corticosteroid treatment was reasoned to be too broad and nonspecific to differentiate between an infectious versus an inflammatory process due to pending infectious workup. In addition, the patient was deemed too sick to risk further decompensation from potential infection. A decision was made by the senior rheumatologist to forego corticosteroids, and narrow treatment specifically targeting non-criteria AOSD. Subcutaneous anakinra 100 mg every eight hours was empirically started for AOSD, given the patient’s critical cardiac decompensation and faltering mental status. He responded with a resolution of chest pain and dyspnea, improved stamina and alertness, and significant improvement of joint pain and joint range of motion after three doses of anakinra. He was weaned off pressor support within 48 hours of anakinra initiation and transferred to medical service.

The patient decompensated two more times. On both occasions, he required intensive levels of care for pressor support. A temporal relationship was observed in both instances between missed doses of anakinra and clinical decompensation 36-48 hours later. On hospital days 11-12, the patient became febrile with a maximum temperature of 39.7°C. By virtue of the high fever, he fulfilled both the Yamaguchi criteria for AOSD as well as the high probability for MAS/HLH by HScore of 70-80% (Table 2) [5-7]. After verifying a positive and compelling response to subcutaneous anakinra 100 mg every eight hours alone, he was empirically initiated on intravenous (IV) hydrocortisone 50 mg every six hours for possible adrenal insufficiency by the ICU team without prior adrenal suppression test. On day 13, he received one dose of subcutaneous methotrexate 25 mg as an adjunct therapy for the treatment of debilitating inflammatory arthritis. His joints responded well though too early to be attributed to the effect of methotrexate. He consequently developed presumptive methotrexate-induced neutropenia with an absolute neutrophil count of 1,400/mcL (RR: 1,700-8,400). He was managed with increased doses of folic acid from 1 mg to 3 mg, cessation of methotrexate, and decreased dosing of anakinra to 100 mg daily. White blood cell count (WBC) nadir at 1.7 x 10^3^/mcL and upward trended to 4 x 10^3^/mcL four days later (Table 1).

Our patient experienced dramatic clinical improvement to combination therapy of anakinra, one dose of methotrexate, and stress dose steroids with hydrocortisone 50 mg every six hours. His fever resolved. His blood pressure (98/62 mmHg-143/90 mmHg) and heart rate (64-88 bpm) normalized in the last five days of admission with a steady decline of inflammatory markers (i.e., CRP from peak 150.1 mg/L to 8 mg/L, RR: <5 mg/L; ferritin down to 2770 ng/mL, RR: 30-400 ng/mL). He experienced significant improvement in joint pain and muscle weakness. He was discharged on day 19 with prednisone 25 mg daily and folic acid 3 mg daily.

In the ambulatory clinic five days after hospital discharge, he endorsed difficulty performing basic activities of daily living and ambulatory dysfunction from worsening painful joint swelling in the wrists, ankles, and feet. He was commenced on cyclosporine 100 mg daily and up-titration of prednisone to 40 mg daily to be tapered by 5 mg each week. Three sent-out serial measurements of soluble interleukin-2 receptor alpha (IL-2R⍺) or soluble CD25 (sCD25) increased by 3.2-7 fold (7,401 pg/mL, 13,209 pg/mL, and 6,069 pg/mL; RR 532-1,891) throughout the hospital course (Table 1). He fulfilled the 2016 classification criteria for MAS given more than 3.2-7 fold normal levels of sCD25 and IL-2R⍺ (Table 2) [5]. Insurance prior authorization for canakinumab was initiated for the management of AOSD and MAS. At the six-week follow-up, he continued to improve on cyclosporine with prednisone tapered down to 15 mg daily, and his first dose of canakinumab. He did not experience recurrent seizures or symptoms of myocarditis. His WBC, Hgb, and CRP normalized, and hyperferritinemia improved to 576 ng/mL (Table 1).

## Discussion

AOSD is a diagnosis of exclusion. We completed an extensive work-up to rule out autoimmune, infectious, and malignant conditions before establishing the diagnosis of AOSD. Several classification criteria for AOSD have been developed, but the Yamaguchi classification criteria are most utilized [7]. The mortality rate of patients with AOSD increases fivefold if AOSD is complicated by MAS [8]. MAS is a subset of secondary HLH associated with rheumatologic disorders. MAS accounts for 10-15% of cases with complicated AOSD [5,8]. Characteristics of MAS are very similar to those of AOSD, which makes early diagnosis particularly challenging.

We suspect that the patient was already manifesting early signs and symptoms of AOSD-MAS during his first hospital admission with seizure, organomegaly, leukocytosis, and hyperferritinemia. We maintained a high index of suspicion for AOSD-MAS based on the constellation of polyarthritis, lymphadenopathy, hepatosplenomegaly, negative ANA/RF, and marked hyperferritinemia of >40,000 ng/mL. Our patient initially presented with atypical features of AOSD, which include recurrent seizures, a pruritic, hyperpigmented, and desquamating rash as opposed to the classic salmon-colored rash, as well as the absence of fever. Unfortunately, a skin biopsy was not performed. He subsequently developed unexpected acute RV failure with biopsy-proven lymphocytic myocarditis. Anakinra was empirically started on hospital day five at the point of right heart decompensation and a deteriorating level of consciousness when our index of suspicion for AOSD and MAS was high but results of BM biopsy, LN biopsy, and soluble IL-2 receptor alpha (sIL-2Rα or sCD-25) were not yet available. The rationale for initiating anakinra first as opposed to corticosteroids was due to its safety profile and its extremely targeted therapeutic effects. Only a selected number of disorders are strongly responsive to IL-1 inhibition, mainly autoinflammatory syndromes, including periodic fever disorders, AOSD, MAS/HLH, and gout. In other words, a strongly positive response to anakinra may clinch the diagnosis without sacrificing patient safety when timely intervention is warranted. The patient did not fulfill the Yamaguchi classification criteria for AOSD until day 11 when he developed a high fever after a lapse of anakinra treatment. The Yamaguchi criteria for AOSD has a sensitivity of 96.2% and specificity of 92.1%, meaning nearly 4% of AOSD patients are missed [7]. A few days after AOSD was firmly diagnosed, he developed leukopenia, hypertriglyceridemia, and sIL-2Rα returned elevated with a calculated HScore of 70-80% probability for MAS/HLH.

We illustrated a rare and atypical case of AOSD presenting with seizures followed by PAH and myocarditis. Literature reported six cases of AOSD that clinically manifested with seizure [4]. These were reported in the context of unknown etiology, liver failure, encephalopathy, and erosion of sella turcica [4]. The seizure was determined to be convulsive syncope secondary to severe anemia in our patient initially by a neurology consultant. However, we cannot be certain if AOSD also played a part in the induction of seizures. In addition, our patient developed PAH with PAP of 55-60 mmHg and acute RHF with moderate tricuspid regurgitation. Group I PAH is a known complication of AOSD [9]. However, in our patient, we were unable to determine the pulmonary hypertension subtype since an extensive workup, such as a V/Q scan, was not performed. Cardiac complications in AOSD have been increasingly recognized and appear to be more prevalent. Based on a recent retrospective chart review of 96 patients, cardiac involvement is present in 29% of AOSD, most commonly pericarditis [2]. It has also been suggested that cardiac involvement is a catalyst of clinical progression in AOSD and is associated with the intensity of inflammatory serum levels [2]. Our patient’s acute cardiac decompensation was observed in conjunction with a peak of hyperferritinemia. Cardiac muscle biopsy revealed lymphocytic infiltrates, suggesting that myocarditis was the underlying cause of acute RHF. MAS triggered an overactivation of T lymphocytes and macrophages leading to a ‘’cytokine storm.” This may have caused persistent hypotension and acute RHF from cardiogenic shock. Based on a small sample size of four, myocarditis associated with AOSD was resistant to corticosteroids alone or in combination with methotrexate in 50% of patients [9]. Similarly, in a review of 48 cases of AOSD-associated myocarditis, only two deaths resulted after steroid-only treatment [10]. Favorable outcomes were observed in all 14 cases treated with steroids and methotrexate, with or without additional biological disease-modifying anti-rheumatic drugs [10]. Anakinra was effective both as first-line and refractory therapy in 100% of the 12 total cases reviewed [2,10].

## Conclusions

In conclusion, we would like to raise awareness of atypical AOSD that may present with seizures and myocarditis. Classification criteria for AOSD do not capture atypical presentations. Clinicians need to maintain a high index of suspicion for non-criteria AOSD, such as delayed onset of fever and transaminitis, and atypical rash in AOSD. The limitation of the HScore for MAS/HLH is that by the time more criteria are fulfilled, the disease may be further along with the greater potential for fatal outcomes. The benefits of anakinra far outweigh the risk of withholding a potentially life-saving treatment for AOSD-MAS. Our case provides further support for the early introduction of interleukin-1 receptor antagonists for patients with AOSD-associated cardiac involvement. Early recognition and prompt initiation of therapy for non-criteria atypical cases of AOSD and MAS may prevent mortality.
